# Analysis of Age-Related White Matter Microstructures Based on Diffusion Tensor Imaging

**DOI:** 10.3389/fnagi.2021.664911

**Published:** 2021-06-28

**Authors:** Yahui Ouyang, Dong Cui, Zilong Yuan, Zhipeng Liu, Qing Jiao, Tao Yin, Jianfeng Qiu

**Affiliations:** ^1^Medical Engineering and Technology Research Center, Shandong First Medical University (Shandong Academy of Medical Sciences), Tai’an, China; ^2^College of Radiology, Shandong First Medical University (Shandong Academy of Medical Sciences), Tai’an, China; ^3^Institute of Biomedical Engineering, Chinese Academy of Medical Sciences & Peking Union Medical College, Tianjin, China; ^4^Department of Radiology, Hubei Cancer Hospital, Tongji Medical College, Huazhong University of Science and Technology, Wuhan, China

**Keywords:** age, gender, diffusion tensor imaging (DTI), tract-based spatial statistics (TBSS), deterministic fiber tracking

## Abstract

Population aging has become a serious social problem. Accordingly, many researches are focusing on changes in brains of the elderly. In this study, we used multiple parameters to analyze age-related changes in white matter fibers. A sample cohort of 58 individuals was divided into young and middle-age groups and tract-based spatial statistics (TBSS) were used to analyze the differences in fractional anisotropy (FA), mean diffusion (MD), axial diffusion (AD), and radial diffusion (RD) between the two groups. Deterministic fiber tracking was used to investigate the correlation between fiber number and fiber length with age. The TBSS analysis revealed significant differences in FA, MD, AD, and RD in multiple white matter fibers between the two groups. In the middle-age group FA and AD were lower than in young people, whereas the MD and RD values were higher. Deterministic fiber tracking showed that the fiber length of some fibers correlated positively with age. These fibers were observed in the splenium of corpus callosum (SCC), the posterior limb of internal capsule (PLIC), the right posterior corona radiata (PCR_R), the anterior corona radiata (ACR), the left posterior thalamic radiation (include optic radiation; PTR_L), and the left superior longitudinal fasciculus (SLF_L), among others. The results showed that the SCC, PLIC, PCR_R, ACR, PTR_L, and SLF_L significantly differed between young and middle-age people. Therefore, we believe that these fibers could be used as image markers of age-related white matter changes.

## Introduction

Nowadays, population aging has become an international trend. In 2009, the world entered the age of aging (United Nations Department of Economic and Social Affairs, 2011). Histopathological evidence has shown that the degeneration of white matter is an important sign of aging (Marner et al., 2003; Bartzokis, 2004). Also, an atomical studies have shown that with increasing age, the ventricles of normal people expand while the brain parenchyma shrinks. Magnetic resonance imaging (MRI) studies have additionally shown that the structure and function of the human brain displays significant changes in adulthood (Raz and Rodrigue, 2006). Age-correlated studies reveal that the changes in white matter may be much higher than those of gray matter (Miller et al., 1980). Upto 40 years of age, the white matter volume increases and is closely related to the formation of the myelin sheath (Courchesne et al., 2000; Bartzokis, 2004). However, with myelin sheath and axon degeneration, the integrity of the white matter changes to varying degrees, with white matter function declining and brain parenchymal structure undergoing atrophy (Bartzokis et al., 2001; Raz et al., 2005; Hedman et al., 2012). A series of such micro and macro changes lead to decline in the cognitive function of the elderly, which can result in an increase in the number of patients with mild cognitive impairment and Alzheimer’s disease. In Alzheimer’s disease white matter volume is much higher than in healthy elderly people (Semendeferi et al., 2002), while the integrity of white matter is lower (Mayo et al., 2018).

Diffusion tensor imaging (DTI) can measure changes in white matter structure related to normal aging by analyzing the diffusion of water molecules in tissues (Pierpaoli and Basser, 1996). The DTI indices of fractional anisotropy (FA), mean diffusivity (MD), axial diffusivity (AD), and radial diffusivity (RD) are often used to assess the structure of white matter fibers (Westlye et al., 2010; Wu et al., 2011a, b; Lamar et al., 2014). Different parameters reflect different aspects of white matter microstructure. FA is a measure of the total directivity of water molecules, which can be used to characterize the ability of water molecules to diffuse along white matter fiber bundles (Basser and Pierpaoli, 2011). The FA value of the parallel fibers of the corpus callosum, for instance, decreases when the fiber bundle is destroyed or interrupted, as in Alzheimer’s disease (Asaad and Lee, 2018; Hao et al., 2020). MD is the average diffusion degree of water molecules. An increase in the MD value indicates a decrease in the free diffusion of water molecules, which is a manifestation of brain degeneration (Rogalski et al., 2012). FA and MD provide information about changes in the diffusion barrier to water molecules. AD measures the diffusion coefficient increase in reflecting axonal degeneration. An increase in RD perpendicular to white matter fibers is related to demyelination (Alves et al., 2015), whereas a decrease in AD reflects ischemic white matter damage (Song et al., 2002). Previous studies on TBSS have focused on changes in FA and MD with age. Many studies have shown that with increasing age, white matter FA decreases, including such white matter areas as the corpus callosum, deep frontal, inferior frontal, medial orbital, fornix, anterior limb of internal capsule, external capsule, anterior cingulum, inferior longitudinal fasciculus, and cerebellar tracts (Madden et al., 2004; Salat et al., 2005; Inano et al., 2011). It has also been shown that FA values for the splenium of the corpus callosum, the corona radiata, the posterior limb of internal capsule, the superior longitudinal fasciculus, and the bilateral superior cerebellar peduncles increase with age (Inano et al., 2011; Houston et al., 2019).

TBSS analysis combines the advantages of whole-brain voxel-based analysis (VBA) analysis and region of interest (ROI) analysis for sampling centrosomes in a single space. It projects the volume data to the white matter skeleton, avoids the partial volume effect, solves the problem of image misalignment to a certain extent, and avoids the need for smoothing (Nichols and Holmes, 2002; Bach et al., 2014). Previous studies (Bartzokis et al., 2003, 2004) have shown that Alzheimer’s disease and mild cognitive impairment occur mostly in the elderly and that decomposition of myelin is the underlying etiological factor contributing to decomposition. Amyloidosis of white matter fiber deposition occurs earlier than typical clinical manifestation. Hence, white matter microstructure changes in middle-age research subjects may enable prediction of clinical symptoms in advance. Accordingly, our research is focused on detecting white matter changes in the microstructure of young and middle-age subjects.

The purpose of this study was to explore the relationship between white matter microstructure and age and gender. Specifically, TBSS analysis based on the white matter skeleton was used to study the relationship between the FA, MD, AD, and RD parameters and age and gender in healthy adults. Significant difference regions obtained from the TBSS analysis were identified as regions of interest and the fiber tracking method was used to study the correlation between fiber number and fiber length with age and gender.

## Materials and Methods

### Participants

A total of 58 subjects aged 20–64 years were recruited, including 39 females and 19 males. The subjects were divided into two cohorts based on age: a youthful group (20–35 years) and a middle-age group (36−64 years). The demographic characteristics of the participants are shown in Table 1. Our data were obtained from Hubei Provincial Cancer Hospital (Wuhan, China). Before the (MR) examination was approved by the Institutional Review Board, a written informed consent was obtained from the subjects. The inclusion criteria were as follows: (1) no family history of mental illness; (2) no use of psychotic drugs within the past 3 months; (3) no contraindications of MRI (such as claustrophobia or pacemaker installed)’ (4) no image quality artifacts; and (5) no history of brain injury or surgery.

**Table 1:** Demographic information of the two groups.

	Young-aged group (20–35)	Middle-aged group (36–64)	χ^2^	*P*
Age (years)	27.46 ± 3.72	45.83 ± 4.82	n.d.	n.d.
Number	24	34	n.d.	n.d.
Gender(F/M)	15/9	24/10	0.418	0.518

*Age is expressed as mean ± SD. Number, the number of participants. n.d., not done*.

### Data Acquisition

Image data were collected with a 3.0T Siemens Skyra MRI, with an 8-channel head coil for reception of radio frequency (RF) signals. The subjects wore professional anti-noise earplugs to blockout noise and used bilaterally affixed sponge pads to reduce head movement artifacts. The DTI data of each subject consisted of one non-diffusion-weighted and 64 diffusion-weighted images acquired with a *b*-value of 1,000 s/mm^2^ uniformly distributed across 64 gradient directions. The parameters were as follows: sequence variant = SK\SP, slice thickness = 4 mm, repetition time (TR) = 5,000 ms, echo time (TE) = 98 ms, flip angle = 90°, acquisition matrix = 128*128, FOV = 1,344*1,344. The parameters for the T1WI analysis were set as follows: slice thickness = 1 mm, TR = 2,300 ms, TE = 2.26 ms, acquisition matrix = 256*256, flip angle = 8°, and FOV = 256*256.

### Data Processing

#### Preprocessing

Diffusion tensor imaging was preprocessed by DTIFit software obtained from the FMRIB diffusion Toolbox (FDT, part of FSL). Data preprocessing adhered to the following protocol. First, the data were inspected for serious artifacts or obvious data loss and discarded if these were detected. Second, the DICOM image was processed by conversion of dcm2nii^1^ to 4D Nifti data. Third, the FSL^2^ taken from DTI data of 58 subjects was subjected to affine registration of non-diffusion volumes (*b* = 0) to correct for head move out and eddy currents. Fourth, the scanning gradient direction was corrected, and then a brain bet was performed. Fifth, the diffusion tensor was fitted to each voxel of the brain mask to obtain FA, MD, AD, and RD diffusion tensor maps.

#### TBSS Analysis

TBSS was used to assess the correlation of FA with age between the two different age cohorts. FA images were aligned with the FMRB158_FA template, and FA images of individual white matter were registered to the FMRIB58_FA template by means of FNIRT and naturalization to obtain all_FA_skeletonised. Then the average FA map and white matter skeleton were constructed based on the total FA registration of the standard space. The mean FA image was generated subsequent to registration and the threshold was set to FA >0.2 in order to exclude the effects of voxels containing gray matter and cerebrospinal fluid. The MD, AD, RD data were also aligned with the MNI 152 space and projected onto the mean FA skeleton by means of projection vectors obtained using the FA data. The FSL randomize command (with 5,000 permutations) was used to generate the statistic maps, threshold free cluster enhancement (TFCE) thresholding was used by statistical inference. The significance level was set at *p*-value < 0.05 with full correction for multiple comparisons (Nichols and Holmes, 2002). White matter fiber bundle information exhibiting significant area differences were projected on to the JHU-ICBM-DTI-81 template. In addition to the FA data from the white matter skeleton of the subjects, TBSS analysis was used to process MD, AD, and RD data obtained from the same voxels. For clusters exhibiting significant differences in FA, MD, AD, and RD between young and middle-age cohorts, we correlated the average FA, MD, AD, and RD of all voxels in clusters which displayed significant differences with age, so as to intuitively study the age-dependent distribution of white matter microstructure parameters.

#### Deterministic Fiber Tracking

The results of preprocessing the fifth step were imported into the DSI-studio^3^ for DTI reconstruction. ROI regions from the TBSS analyses were imported into the DSI-studio for fiber tracking. As per our previous reports (Liu et al., 2018), the fiber tracking parameters were set as follows: FA was 0.15, the step size was 0.47, and the angular threshold was less than 60°. Additionally, tract lengths were restricted to 30–300 mm in length.

### Statistical Analysis

A Chi-square test was performed to evaluate differences between genders within and between groups. Spearman analysis was used to study the correlation between age and average white matter microstructure parameters (FA, MD, AD, RD) of all voxels in clusters with a significant difference with age. A *p*-value < 0.05 was selected to indicate a statistically significant difference and age-related trends were delineated with a scatter diagram. For deterministic fiber tracking, spearman analysis was used to study the relationship between fiber length, fiber number, and age. Spearman analysis results were corrected by FDR. In addition, Spearman was used to analyze correlation between FA, MD, AD, RD, fiber length and age in the two subgroups of young group, middle-age group respectively. With *p*-values < 0.05 indicating a statistically significant difference.

## Results

### Correlation Between TBSS Analysis White Matter Microstructure and Age

**Figure 1:**
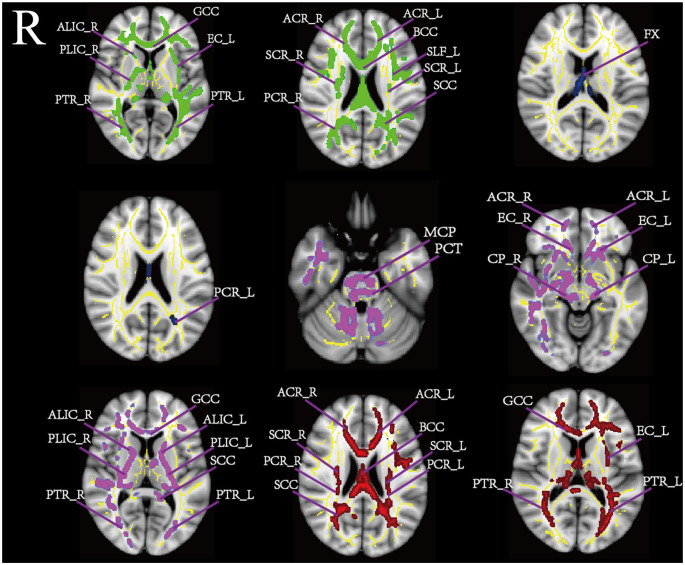
This figure shows the significant different regions of tract-based spatial statistics (TBSS) under the influence of age based on MNI_152 template distribution. The yellow area is the white matter skeleton template, fractional anisotropy (FA;green), mean diffusion (MD; blue), axial diffusion (AD; purple), and radial diffusion (RD; red).

We performed a voxelwise comparison of FA, MD, AD, and RD values displaying age related difference between the two cohorts. The results are shown in Table 2. Figure 1 shows the significant differences between young and middle-age group in the TBSS analysis. The FA of the middle-age group was lower than that of the younger group (*p* < 0.01) in numerous WM regions including the GCC, SCC, PCR_R, SCR, PLIC_R, BCC, ACR, ALIC_R, PTR, and EC_L. We did not observe an increase in age-related FA. The AD in many brain regions, including the MCP, ACR, PCT, CP, GCC, SCC, PLIC, EC, and ALIC, was significantly lower in the middle-age than in the younger group. By contrast, the middle-age group had MD and RD values significantly higher than those of the young group (*p* < 0.001). The MD differential region was FX, PCR_L. Higher RD values for the middle-age group were observed in the GCC, SCC, BCC, ACR, SCR, PCR, EC_L, and PTR.

**Table 2:** The TBSS results were obtained by age groups.

	Region
FA Middle age< Young people	Left external capsule Anterior corona radiata Superior corona radiata Body of corpus callosum Genu of corpus callosum Splenium of corpus callosum Right posterior corona radiata Left superior longitudinal fasciculus Right posterior limb of internal capsule Right anterior limb of internal capsule Posterior thalamic radiation (include optic radiation)
MD Middle age > Young people	Fornix (column and body of fornix) Left posterior corona radiata
AD Middle age< Young people	External capsule Cerebral peduncle Anterior corona radiata Genu of corpus callosum Middle cerebellar peduncle Splenium of corpus callosum Anterior limb of internal capsule Posterior limb of internal capsule Pontine crossing tract (a part of MCP) Posterior thalamic radiation (include optic radiation)
RD Middle age > Young people	Left external capsule Body of corpus callosum Anterior corona radiata Superior corona radiata Posterior corona radiata Genu of corpus callosum Splenium of corpus callosum Posterior thalamic radiation (include optic radiation)

**Figure 2:**
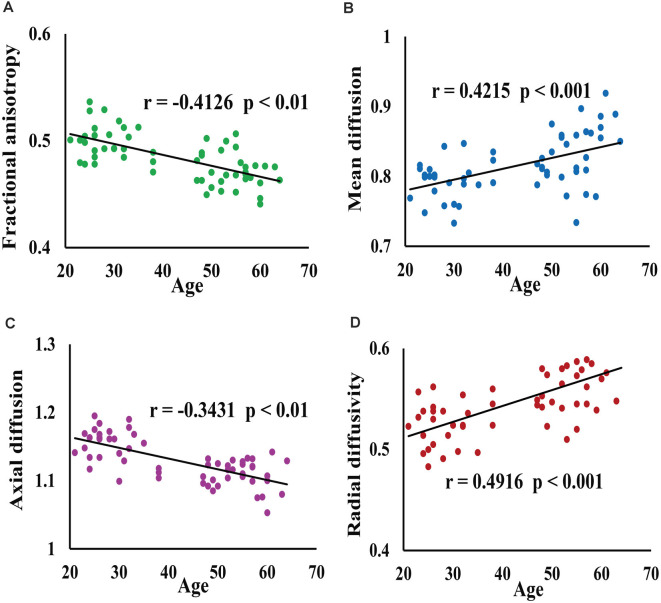
This figure shows the results of correlation between age and average white matter microstructure parameters (FA, MD, AD, RD) of all voxels in clusters. Panel **(A)** represents FA value, **(B)** represents MD value, **(C)** represents AD value, **(D)** represents RD value; where **(B)**, **(C)** and **(D)** ordinates expand 10^3^ times. The scatter plot of FA **(A)** and AD **(C)** of voxels with age showed that age was negatively correlated with FA and AD, while the scatter plot of MD **(B)** and RD **(D)** with age showed that age was positively correlated with MD and RD.

The scatter plots of FA, MD, AD, and RD of all voxels in the cluster with a significant difference between young and middle-age groups were shown in Figure 2. Spearman analysis revealed that FA and AD were significantly negatively correlated with age (*p* < 0.01). By contrast, MD and RD were significantly and positively correlated with age (*p* < 0.001).

Spearman was used to analyze correlation between FA, MD, AD, RD and age in the two subgroups of young group, middle-age group. As shown in Supplementary Table 1.1 and Supplementary Figure 2.1, it was found that FA, MD, AD, RD in the young group and FA, AD in the middle-age group had no significant correlation with age. Whereas, in the middle-age group, MD and RD were significantly positively correlated with age.

### Correlation Between TBSS Analysis White Matter Microstructure and Gender

No significant gender differences were found in the TBSS analysis.

### Correlations Between Deterministic Fiber Tracking With Age in Regions of Interest

**Figure 3:**
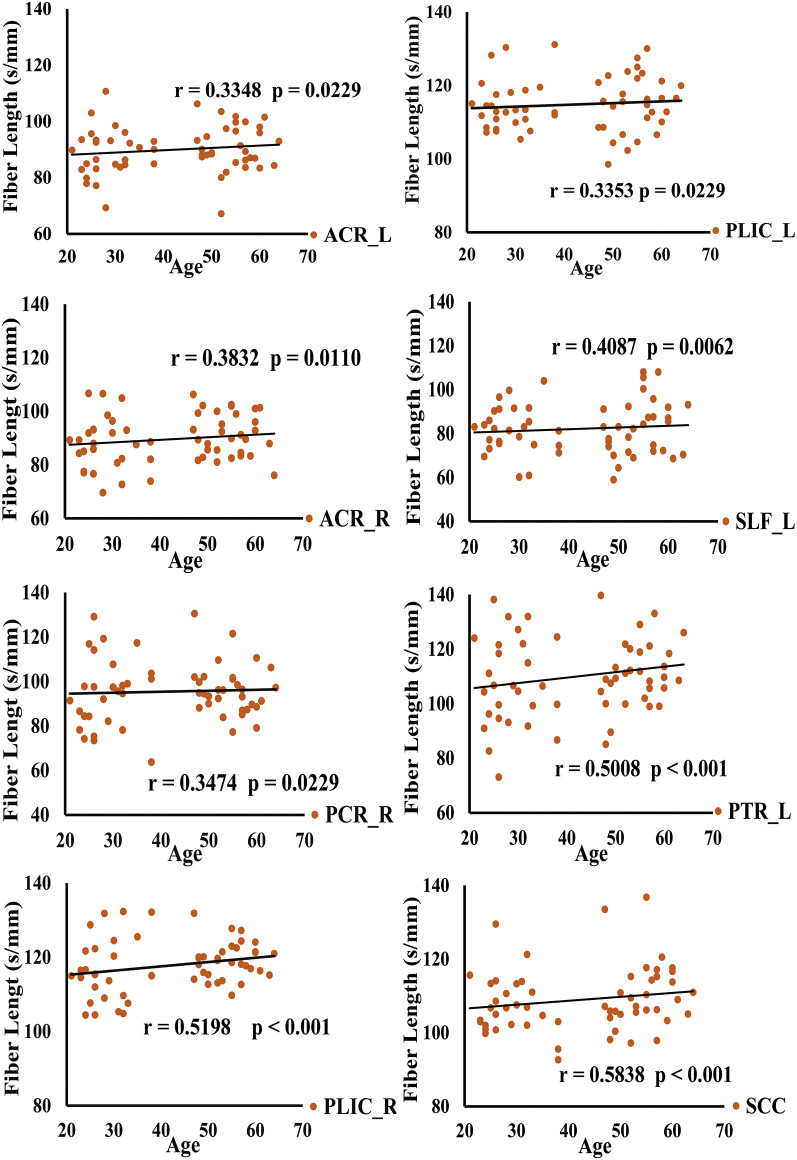
Spearman correlation analysis of the relationship between fiber length and age is shown in this figure.

**Figure 4:**
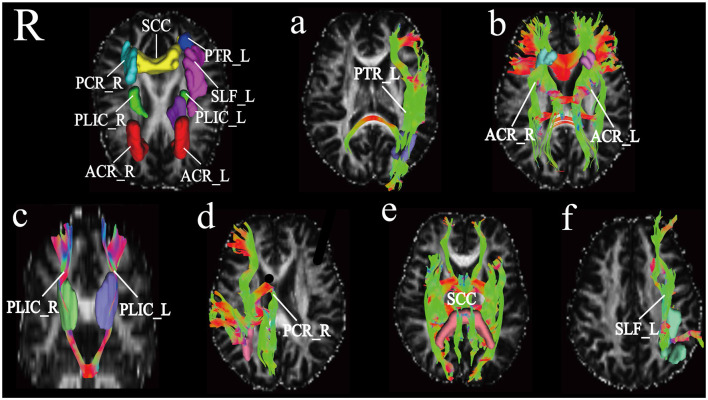
Based on the JHU template, differential white matter fibers obtained from deterministic fiber trackingPanel **(A)** represents the white matter fiber PTR_L [the left of posterior thalamic radiation (include optic radiation)], **(B)** white matter fibers shown are ACR (anterior corona radiata), **(C)** white matter fibers shown are PLIC (posterior limb of internal capsule), panel **(D)** shows fibers as PCR_R (right posterior corona radiata), **(E)** SCC (splenium of corpus callosum), (F) SLF_L (left superior longitudinal fasciculus), respectively are shown in this figure.

DSI-studio was used to track the fibers in different regions obtained by TBSS analysis and measure the number and length of fibers in each region. Spearman analysis revealed that the number of BCC fibers increased with age, while the number of CP_L fibers significantly decreased with age. After FDR verification, no significant correlation between fiber number and age was observed. However, we observed a significant positive correlation between age and fiber length in PTR_L, SLF_L, ACR_L, ACR_R, SCC, PCR_R, PLIC_L, and PLIC_R (Table 3). Their trends are shown in the scatter diagrams of Figure 3. The specific white matter fibers associated with age are shown in Figure 4.

**Table 3:** The deterministic fiber tracking results were obtained by age.

	**Fiber Number**		**Fiber Length**	
**ROI**	*p*-Value	*r*	*p*-Value	*r*
ACR_L	0.6148	0.1216	0.0229*	0.3348
ACR_R	0.7854	0.0759	0.011**	0.3832
ALIC_L	0.0556	−0.3542	0.3508	0.174
FX	0.2735	−0.2188	0.6626	0.0848
GCC	0.2735	−0.2056	0.6626	0.0817
PCR_R	0.2735	0.241	0.0229*	0.3474
BCC	0.0566	−0.3353	0.8144	0.0447
PTR_L	0.2735	0.2308	<0.001***	0.5008
SCC	0.7854	0.0786	<0.001***	0.5838
SCR_L	0.2735	−0.2041	0.4620	0.14
SCR_R	0.8600	0.0277	0.1492	0.2359
SLF_L	0.0556	0.3521	0.0062**	0.4087
CP_L	0.0556	−0.3353	0.8445	−0.0329
CP_R	0.6455	−0.1101	0.6626	0.0941
EC_L	0.8600	0.0507	0.3935	0.1592
EC_R	0.8600	0.0553	0.8144	−0.0444
MCP	0.8600	−0.0252	0.6626	0.099
PCT	0.4671	−0.152	0.8543	0.0247
PLIC_L	0.8600	−0.0237	0.0229**	0.3353
PLIC_R	0.8600	0.0436	<0.001***	0.5198

Spearman correlation efficient. FDR correction was used for correlation analysis. *Corrected *p*-value < 0.05. **Corrected *p*-value < 0.01. *** Corrected *p*-value < 0.001. L, left; R, right. GCC, Genu of corpus callosum; PCR, Posterior corona radiata; EC, External capsule; PTR, Posterior thalamic radiation (include optic radiation); CP, Cerebral peduncle; ACR, Anterior corona radiata; ALIC, Anterior limb of internal capsule; BCC, Body of corpus callosum; FX, Fornix (column and body of fornix); PLIC, Posterior limb of internal capsule; SCC, Splenium of corpus callosum; SLF, Superior longitudinal fasciculus; MCP, Middle cerebellar peduncle; PCT:,Pontine crossing tract (a part of MCP); SCR, Superior corona radiate.

Spearman analysis showed that ACR fiber length in middle-age group and ACR, PLIC, SLF_L fiber length in young group had no significant correlation with age. PCR_R, PTR_L, SCC in young group, and PLIC, SLF_L, PCR_R, PTR_L, SCC in middle-age groups fiber length were significantly positively correlated with age. The specific statistical analysis and results are shown in Supplementary Table 1.2 and Supplementary Figure 2.2.

## Discussion

### Correlation Between White Matter Microstructure and Age: TBSS Analysis

TBSS analysis was performed to study the age-related white matter diffusion parameters. In numerous WM regions FA values obtained from the middle-age cohort was decreased with respect to those obtained in the younger group (*p* < 0.01). For instance, GCC, BCC, SCC, PCR_R, SCR, PLIC_R, ACR, and PTR. AD was significantly lower in the middle-age than in the young people, such as MCP, ACR, PCT, CP, GCC, SCC, PLIC, EC, and ALIC. The MD differential region was FX, PCR_L. And in terms of RD, the parts of the middle-age group higher than the young people (*p* < 0.001) including GCC, SCC, BCC, ACR, SCR, PCR, EC_L, and PTR.

Previous studies on age-related DTI have confirmed that there is a significant correlation between decreased FA and increased RD (Davis et al., 2009; Madden et al., 2009; Zhang et al., 2010). In our research, it was found that FA in EC_L, ACR, SCR, PTR, and PCR_R regions decreased, while RD value increased, which was consistent with previous research conclusions. A reduction in the FA value is indicative of white matter microstructure degradation with age. While an increase in brain water content can influence this value, the changes in RD values in some brain areas appear to have been mainly due to demyelination and fiber thinning.

In terms of function, the volume of anterior white matter was closely related to FA, while the function of frontal area decreased significantly with age (Cabeza et al., 2004). FA in the frontal white matter decreases with age, which affects the connection of the frontal lobe, which is an important sign of atrophy. There was consistent with the principle of age-related cognitive impairment (Hugenschmidt et al., 2008). The decrease of FA in the corona radiate, internal capsule, and anterior thalamic radiation, particularly, indicates that the connection between the thalamus and frontal lobe was impaired. Physiologically, the decrease of FA in white matter is considered to be an age-related change in myelin integrity and structure. The observation that the FA was positively correlated with white matter volume, suggests that FA may be a “microstructural index” of volume loss, which could be used as a sensitive marker to detect changes of white matter microstructure in a trophic tissues. Consistent with the change in FA values we observed an increase in the age-related RD (Song et al., 2003). The increase in RD suggests that demyelination is a likely contributing factor to age-related white matter abnormalities (Pierpaoli et al., 2001; Song et al., 2002, 2003). Myelin degradation exposes axonal regions that do not have sufficient ion channels, leading to slow action potential propagation until more ion channels can be obtained when the myelin is fully restored (Hugenschmidt et al., 2008). Animal experiments have shown, for instance, that more oligodendrocytes are formed by the myelin sheath in the corpus callosum than in the cerebral cortex in adult mice and that changes in the myelin sheath alter the plasma channels of the myelin tablet and cause myelin pore fragmentation (Peters, 2002).

We found that the FA and AD values in GCC, BCC, and SCC regions of middle-age people were lower than that of young people, while the RD value of middle-age people was significantly higher than that of young people. AD provides relevant information about axonal integrity or spatial changes associated with extra axonal cells. Compared with young people, the value of AD in multiple white matter areas in the middle-age group decreased, which reflected damage to the macrostructure of these brain regions in middle-age people involving damage to axon integrity or axon contraction, which may be magnified with age. Clinical and practical studies have shown that axons and myelin sheaths of myelinated fibers in many white matter tracts are associated with age-related degeneration (Bowley et al., 2010).

The corpus callosum is a highly organized bundle of fibers connecting the left and right hemispheres of the brain, in which the fiber bundle composition is differentiated. The SCC and GCC are composed of a high-density fine fibers, while the corpus callosum is composed of a high proportion of large-diameter fibers (Aboitiz et al., 1992). The SCC is the connective fiber between the hemispheres, which mainly connects the visual area with the parietal lobe and the posterior cingulate band (Voineskos et al., 2012). The anterior part of the myelin sheath consists of fine late myelin fibers extending from the junction of the parietal and temporal lobes. The posterior part, composed of thick early myelin fibers, connects the visual area (Dougherty et al., 2005; Saenz and Fine, 2010) and the central fibers connect the dorsal visual area and the combined parietal region (Putnam et al., 2010). A study of 20–85 year-old subjects found that aging had a small effect on the corpus callosum volume (Bachman et al., 2014) and that the SCC plays an important role in young people’s intellectual function increases with age (Knyazeva, 2013). Other clinical studies have shown that age-related degenerative changes in the white matter pathway through the corpus callosum can lead to functional defects in both hands. It has also been reported that the microstructure of the SCC is closely related to a decrease in hand-to-hand coordination with aging (Sullivan et al., 2001). Consistent with these observations, damage or thinning of the corpus callosum is associated with reduced performance, attention, working memory, language fluency, and memory tests (Redmond et al., 2018).

### Deterministic Fiber Tracking With Age in Region of Interest

There was no significant correlation observed between the number of fibers in the white matter regions and age. However, we found that the length of fibers in the ACR, SLF_L, PCR_R, PTR_L, SCC, and PLIC regions increases with age. These results are generally consistent with some other observations obtained in our lab, which show that the fiber lengths in SCC, PCR, and PTR regions increase with age (Liu et al., 2018).

The SLF connects the temporal parietal language area with the ipsilateral frontal lobe and thus plays an important role in the language network. Age-related FA decline patterns of the SLF differ between men and women. In females, the FA exhibits an inverted U change with age, while in males the FA decreases with age (Madhavan et al., 2014). The ACR and PCR are part of the edge-the thalamus-cortical circuits, which includes the span from the internal capsule to the projection of the cortex, the thalamus, and the PLIC, which projects sensory information from the thalamus to the cortex. The thalamus fiber connection is involved in the emotion regulation system and plays an important role in perception and movement. The PLIC plays a role in stroke and in depression (Karababa et al., 2015; Hyett et al., 2018; Kang et al., 2018). The PTR is an important bridge between the associated thalamus and the visual cortex and with maturity improves the quality of movement and visual function (Aeby et al., 2009).

Reports of the total length of myelin fiber in young adults range from 150,000–180,000 km (Pakkenberg et al., 2003), but decrease by 45% between the age of 20 and 85 (Marner et al., 2003). Moreover, clinical autopsy results confirm that the length of myelinated fibers in the whole brain decreases with age. We hypothesize that the observation of an increase in the length of white matter fibers with age may be due to environmental factors or, due to the small number of subjects, may be a false-positive result. In subsequent studies, we will increase the number of subjects to verify whether our conclusion about the variation of fiber bundle length with age is correct.

When we studied the age correlation of FA, MD, AD, RD, fiber length in the young and middle-age groups, we found that FA, MD, AD, RD in the young group and FA, AD in the middle-age groups had no significant correlation with age. In addition, the fiber length of ACR in the middle-age group and the fiber length of ACR, PLIC, SLF_L in the young group had no significant correlation with age. This may be because after the refinement of the two subgroups, the age range of the subjects in each subgroup was narrowed and the number of subjects was significantly reduced, resulting in no correlation between some diffusion parameters, fiber length in the ROI region and age. However, there are still a number of results that confirm our findings on the age-related relationship between TBSS analysis and deterministic fiber tracking. For example, in the middle-age group, MD and RD was significantly positively correlated with age. The PCR_R, PTR_L, SCC in the young group and the PLIC, SLF_L, PCR_R, PTR_L, SCC fiber length in the middle-age group were significantly positively correlated with age. These conclusions are consistent with the results of our previous studies. Therefore, it also suggests that aging research needs a large number of subjects with a wide enough age range. This will make the results more convincing.

## Limitation

TBSS increases the complexity while increasing the skeletonization steps, which may reduce the overall transparency of the data and conceal the authenticity of the original data (Bach et al., 2014). In addition, TBSS’s excessively rigorous replacement test has obvious disadvantages, which may conceal some positive results and introduce errors. Additionally, TBSS analysis based on FA projection generates errors resulting from the inaccuracy of the FA skeleton projection (Bach et al., 2014), with even the location of some areas is limited to the white matter skeleton, thereby increasing false-negative results. On the other hand, the small number and limited age range of our study’s subjects may lead to false positive results. Further research should include more subjects and a wider age range in order to apply the findings to the general population.

## Conclusion

This study showed that the microstructure of white matter in the brain changes significantly with aging. Compared with middle-age people, FA and AD values obtained from many white matter regions in young people were significantly lower than those observed in middle-age people, whereas MD and RD values in some white matter regions were significantly higher. Spearman correlation between age and the average white matter microstructure parameters FA, MD, AD, and RD obtained from all voxels in the cluster showed a significant difference between the young and middle-age groups, with FA and AD values displaying a significant negative correlation with age (*p* < 0.01). Conversely, MD and RD displayed a significant positive correlation with age (*p* < 0.001). One of the new findings of this study is based on the deterministic fiber tracking that demonstration of a positive correlation between fiber length and age in the ACR, PCR, SLF_L, PTR, PLIC, and SCC regions.

## Data Availability Statement

The raw data supporting the conclusions of this article will be made available by the authors, without undue reservation.

## Ethics Statement

The studies involving human participants were reviewed and approved by Institutional Review Board. The patients/participants provided their written informed consent to participate in this study. Written informed consent was obtained from the individual(s) for the publication of any potentially identifiable images or data included in this article.

## Author Contributions

TY and JQ designed the study. DC and YO performed image processing and wrote the manuscript. ZY and QJ contributed to the statistical analysis. YO, ZL, TY, and JQ reviewed and edited the manuscript. All authors contributed to the article and approved the submitted version.

## Conflict of Interest

The authors declare that the research was conducted in the absence of any commercial or financial relationships that could be construed as a potential conflict of interest.

## Footnotes

^1^http://people.cas.sc.edu/rorden/mricron/dcm2nii.html

^2^https://fsl.fmrib.ox.ac.uk/fsl/fslwiki

^3^http://dsi-studio.labsolver.org/
