# Supplementary material for: Analysis of Age-Related White Matter Microstructures Based on Diffusion Tensor Imaging
Source: Front Aging Neurosci. 2021 Jun 28;13:664911. doi: 10.3389/fnagi.2021.664911 (PMC8273390; doi:10.3389/fnagi.2021.664911)
Supplement: Supplementary file 1 [file Table_1.DOCX]

Supplementary Material

# 1. Supplementary Data

## TBSS analysis

Supplementary Table 1.1. The correlation between FA, MD, AD, RD and age in the two subgroups was analyzed respectively

|  | **Young group** | | **Middle group** | |
| --- | --- | --- | --- | --- |
|  | **p** | **r** | **p** | **r** |
| FA | 0.5561 | -0.1264 | 0.2007 | -0.2250 |
| MD | 0.6540 | 0.0964 | 0.0338* | 0.3651 |
| AD | 0.7121 | 0.0794 | 0.6089 | -0.0910 |
| RD | 0.2736 | 0.2328 | 0.000*** | 0.4916 |

## Deterministic fiber tracking analysis

Supplementary Table 1. 2. Correlation between fiber length of ROI and age in the two subgroups was analyzed respectively

|  | **Young group** | | **Middle group** | |
| --- | --- | --- | --- | --- |
|  | **p** | **r** | **p** | **r** |
| ACR_L | 0.0814 | 0.4114 | 0.4566 | 0.1493 |
| ACR_R | 0.0994 | 0.3783 | 0.0921 | 0.3288 |
| PTR_L | 0.0201* | 0.5352 | 0.0357* | 0.4115 |
| SCC | 0.0058** | 0.6136 | 0.0011** | 0.6278 |
| SLF_L | 0.1465 | 0.3347 | 0.0058** | 0.5245 |
| PCR_R | 0.0201* | -0.5313 | 0.6739 | 0.0749 |
| PLIC_R | 0.1864 | 0.3021 | 0.0058** | 0.5315 |
| PLIC_L | 0.5744 | 0.1321 | 0.0413** | 0.3954 |

# 2. Supplementary Figures


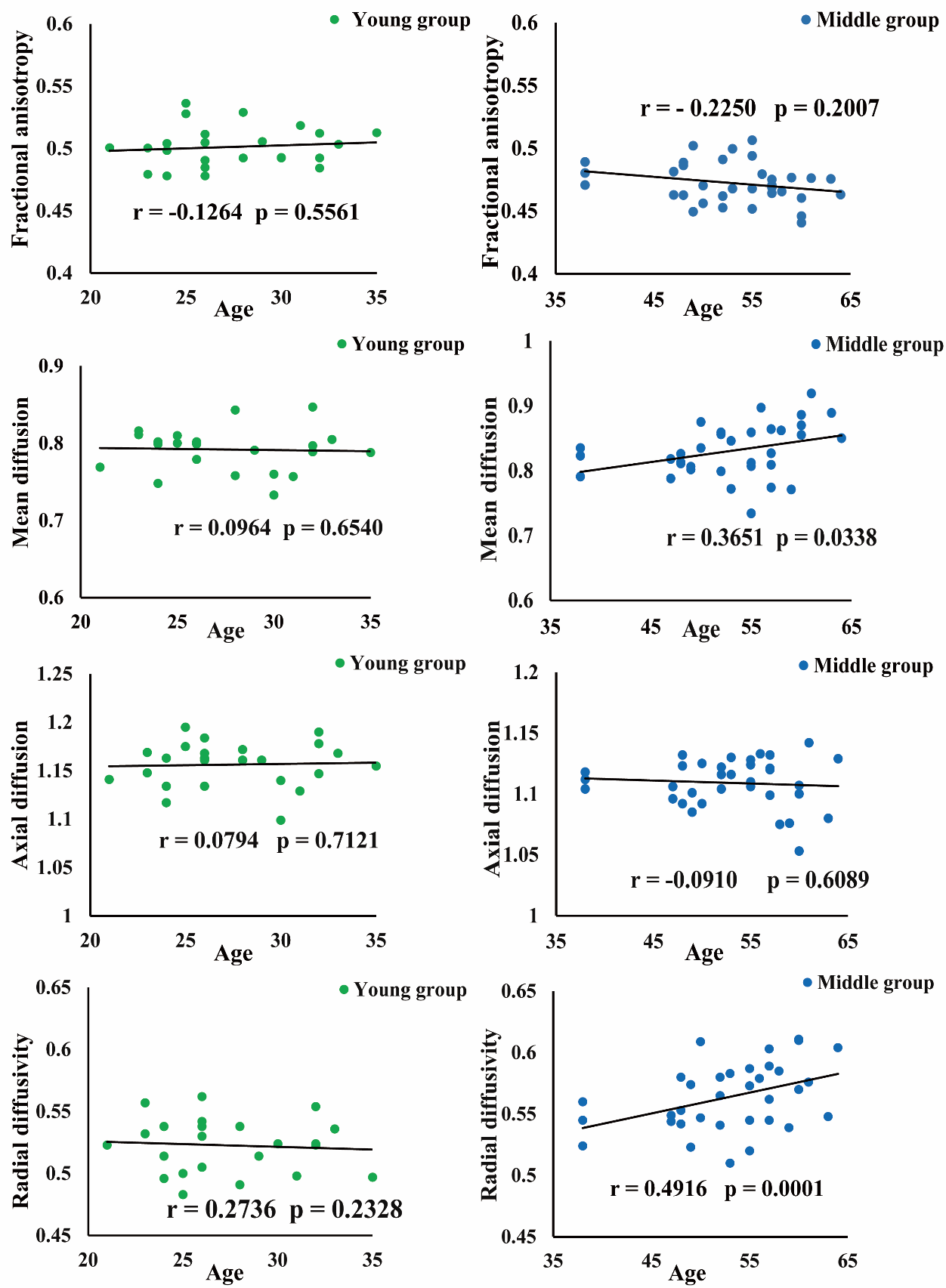


Supplementary Figure 2.1. The correlation of FA, MD, AD and RD with age in the two subgroups was analyzed respectively. (For the convenience of data processing, the ordinate values of MD, AD and RD are magnified 1,000-fold.)


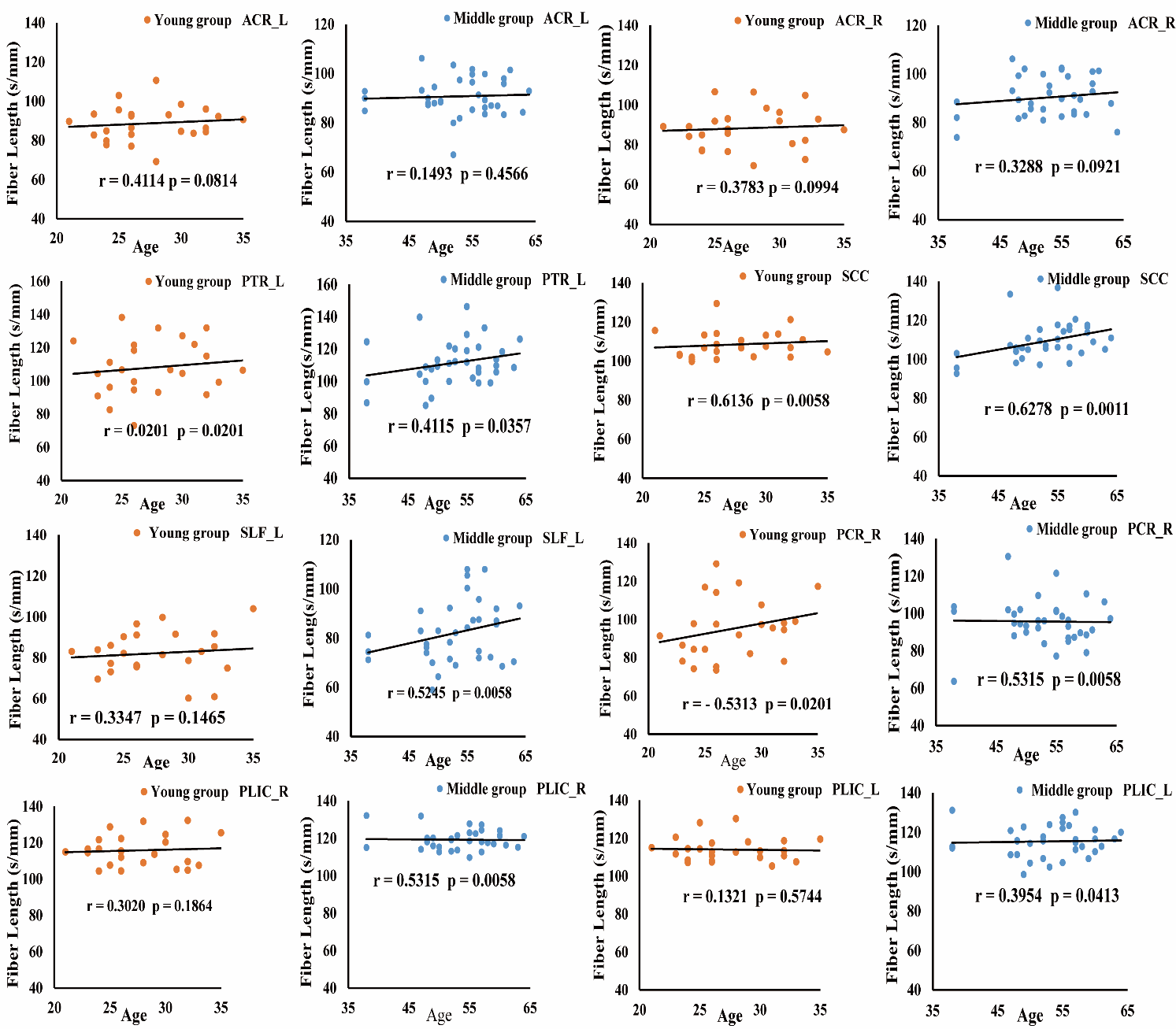


Supplementary Figure 2.2. The figure shows the correlation analysis between the ROI fiber length and age of the two subgroups, respectively.
