# Supplementary figures and images for: Analysis of Age-Related White Matter Microstructures Based on Diffusion Tensor Imaging
Source: Front Aging Neurosci. 2021 Jun 28;13:664911. doi: 10.3389/fnagi.2021.664911 (PMC8273390; doi:10.3389/fnagi.2021.664911)

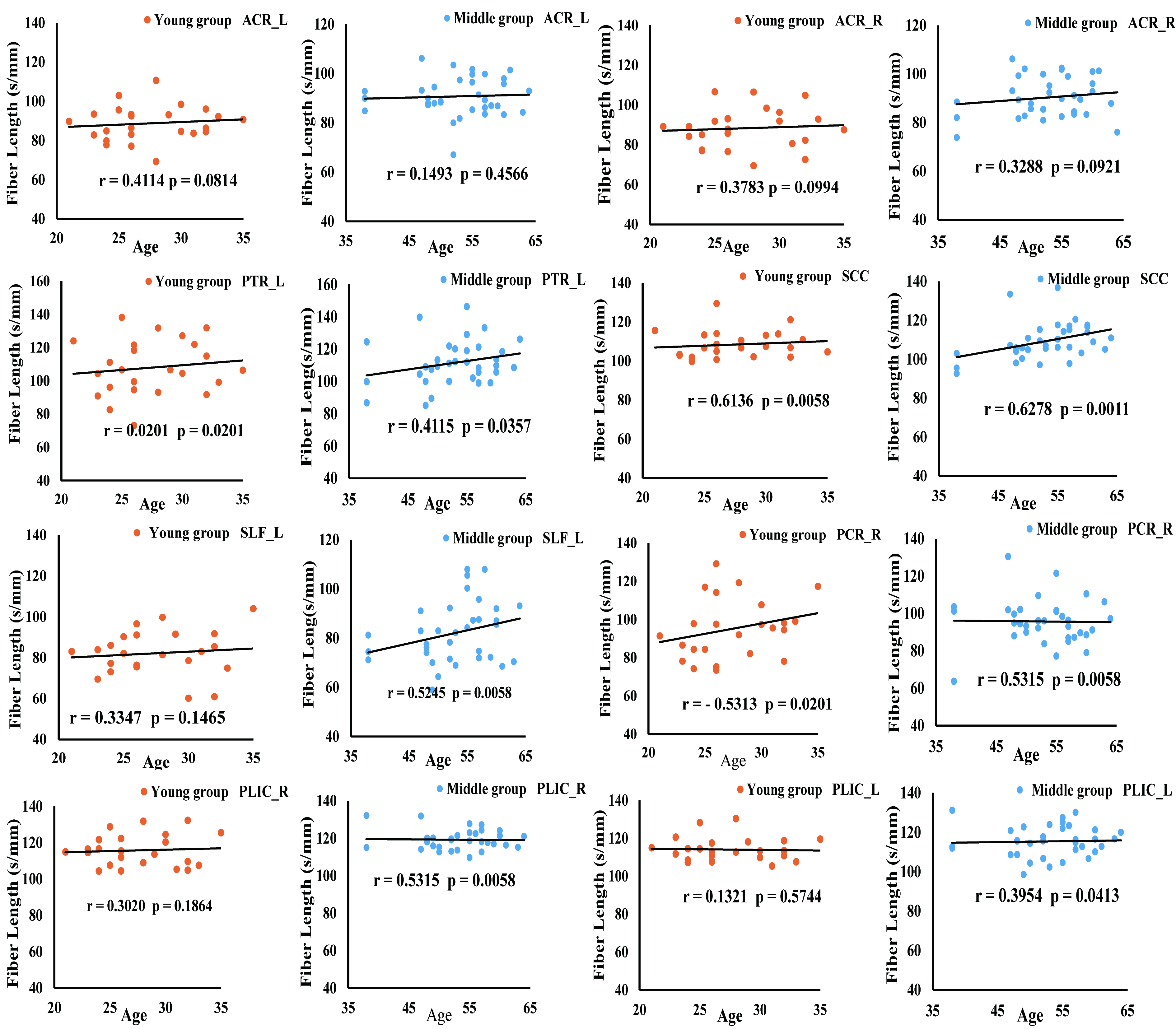

Supplement: Supplementary file 2 [file Image_1.TIF]

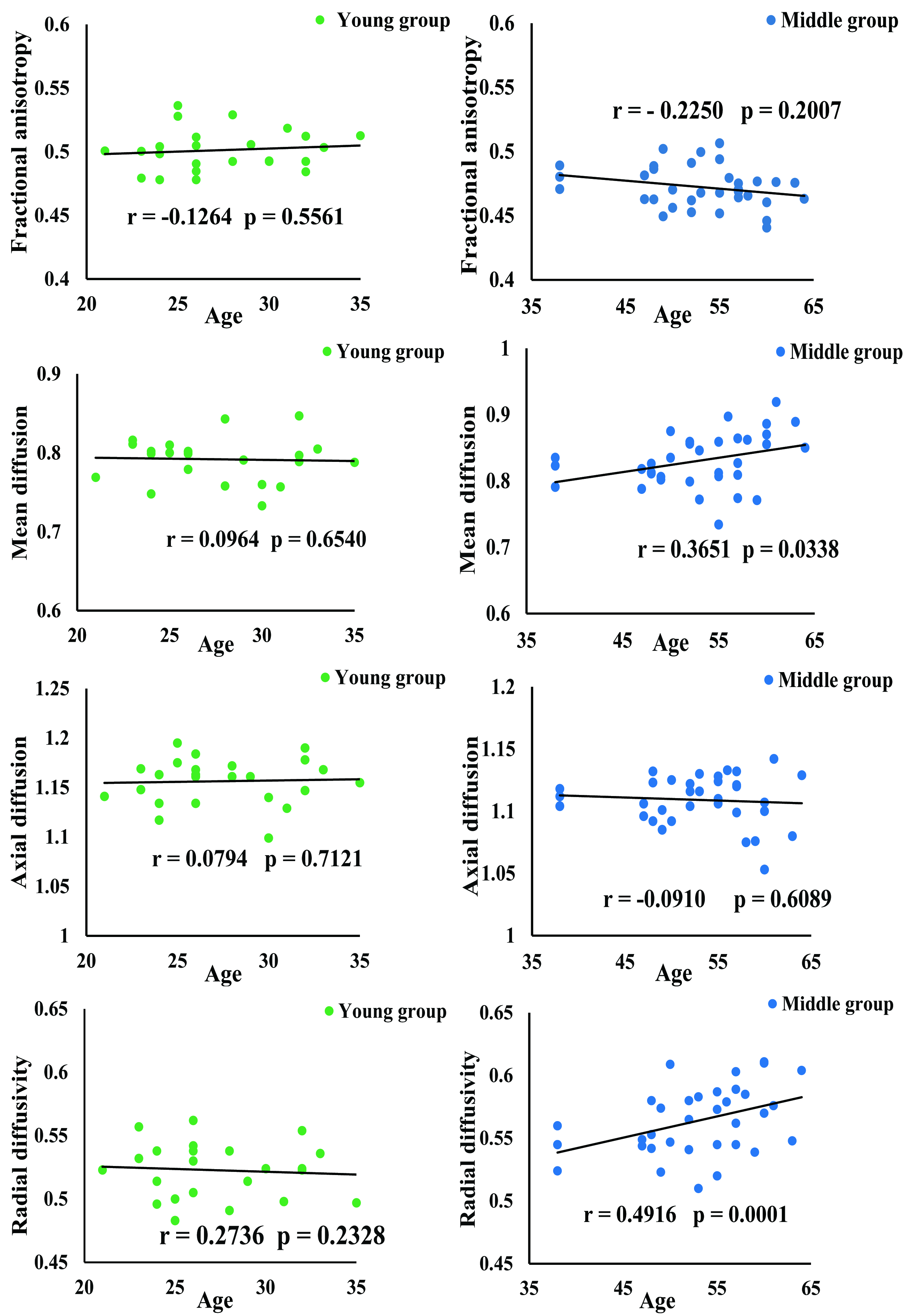

Supplement: Supplementary file 3 [file Image_2.TIF]
